# Intrahepatic Reactive Lymphoid Hyperplasia: A Case Report and Review of the Literature

**DOI:** 10.1155/2018/9264251

**Published:** 2018-09-06

**Authors:** Samantha Seitter, Zachary D. Goodman, Theodore M. Friedman, Timothy R. Shaver, George Younan

**Affiliations:** ^1^Department of Surgery, Inova Fairfax Hospital, Fairfax, VA, USA; ^2^Department of Pathology, Inova Fairfax Hospital, Fairfax, VA, USA; ^3^Department of Pathology, Inova Fair Oaks Hospital, Fairfax, VA, USA; ^4^Division of Hepato-Pancreato-Biliary Surgery, Virginia Surgery Associates, Fairfax, VA, USA

## Abstract

**Introduction:**

Reactive lymphoid hyperplasia (RLH) is a rare and benign lesion found in organs of the gastrointestinal tract, skin, lung, orbit, and more rarely in the liver. Due to its similar appearance on imaging, it is hard to differentiate from primary liver malignancies. The following is a case report of a patient presenting with a suspicious liver lesion found to be RLH associated with primary biliary cirrhosis (PBC), after surgical resection.

**Presentation of Case:**

A 54-year-old woman presented with nonspecific abdominal pain, and her workup included axial imaging of the abdomen that showed a suspicious lesion in her liver. After an extensive workup, which included a percutaneous biopsy, failed to confirm a diagnosis, a laparoscopic surgical resection was recommended.

**Discussion:**

RLH is a rare condition of the liver, presenting in a suspicious fashion and raising concerns for a primary liver malignancy. RLH should be considered in the differential diagnosis of small hepatic lesions in middle-age females in the absence of any significant risk factors for hepatocellular carcinoma (HCC). RLH tends to be associated with PBC of the liver.

**Conclusion:**

RLH of the liver is a rare, hard to diagnose, benign lesion. When intrahepatic, it cannot be easily differentiated from primary liver tumors and frequently requires surgical resection for pathological diagnostic confirmation.

## 1. Introduction

Reactive lymphoid hyperplasia, a proliferation of benign, polyclonal lymphocytes, forming localized mass-like lesions, can present in many organs of the body and mimic primary or secondary malignancies [1]. RLH, originally termed pseudolymphoma or nodular lymphoid lesion, can present in the skin, orbit, lung, spleen, pancreas, stomach, breast, intestines, and very rarely in the liver [2]. On imaging, RLH can resemble hepatocellular carcinoma or malignancies metastatic to the liver, rendering surgical resection the most accurate diagnostic modality [2, 3]. To the best of our knowledge, there have been about fifty-five intrahepatic RLH described in the literature, with a tendency to be found in middle-aged women with autoimmune diseases, where HCC was by far the most common preoperative diagnosis [2, 4, 5].

We present here a case of RLH in the left lateral section of the liver in a 54-year-old woman, requiring a laparoscopic left lateral sectionectomy for final diagnosis.

## 2. Case Presentation

**Figure 1:**
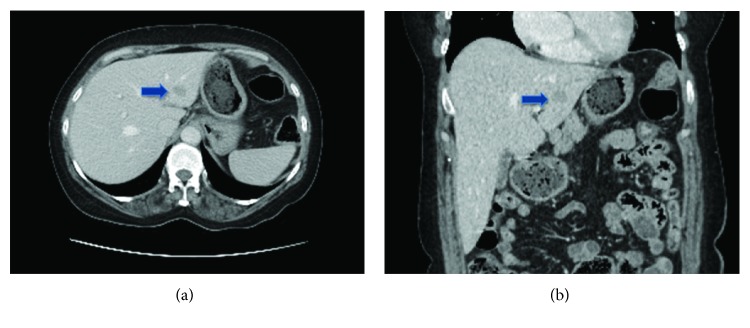
Computed tomography (CT) axial image showing an irregular hypodense lesion in the left lateral section of the liver in (a, blue arrow). The same lesion is shown in the coronal plane in (b, blue arrow).

**Figure 2:**
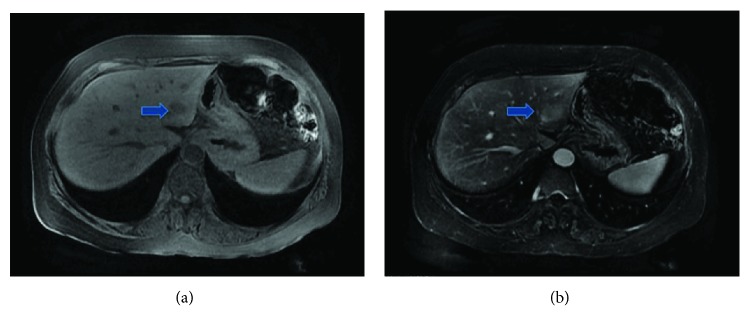
Magnetic resonance imaging (MRI) axial cuts showing the left lateral section liver lesion being hypointense on T1-weighted images in (a, blue arrow) and slightly hyperintense on T2-weighted MRI images in (b, blue arrow).

A 54-year-old Asian American woman presented to her family physician with right side flank pain. She had no other symptoms, and her physical exam was normal. Laboratory testing showed elevated liver function tests including alkaline phosphatase and aspartate aminotransferase. An abdominal ultrasound showed a 1.5 cm hypodense lesion in the left lobe of the liver with associated porta hepatis lymphadenopathy. Computed tomographic (CT) scan revealed a 1.8 × 1.4 cm hypodense mass located in the left lateral section of the liver with minimal peripheral enhancement (Figure 1). Magnetic resonance imaging (MRI) of the liver showed a T1 hypointense and T2 mildly hyperintense lesion with indeterminate enhancement (Figure 2). No other abnormalities were found on axial imaging. A CT-guided biopsy showed nodular collections of polyclonal T and B lymphocytes and plasma cells. Tumor markers, including AFP, CEA, and CA 19-9 were within normal limits, and her hepatitis panel was negative. Serum antimitochondrial antibody (AMA) level was normal. She had normal upper and lower endoscopies.

Based on her clinical presentation, imaging, and an indeterminate pathology report, she was seen at the hepatobiliary high-risk clinic and a laparoscopic left lateral sectionectomy of the liver was recommended. The patient had an uneventful hospital stay postoperatively and was discharged home on postoperative day 3.

Her final pathology revealed nodular reactive lymphoid follicular hyperplasia (RLH) and evidence of primary biliary cholangitis (PBC), which was not diagnosed until final pathology was obtained.

## 3. Discussion

First reported by Snover et al., reactive lymphoid hyperplasia has been reported in many organs in the body and rarely in the liver [6, 7]. More than 50 cases have been described in the English literature since then, with a predilection to middle-aged women (female : male ratio is 8 : 1), with some types of autoimmune disease in about 40% of the cases [8]. Most patients were in their 6th decade of life with an average age at diagnosis of 55 years. Most lesions were solitary, and very few were multifocal (13%). They were generally small in size with average size of 2 cm but can reach large sizes with the largest being around 10 cm [1, 8].

The pathophysiology of RLH of the liver is not well understood. It was thought that reactive immune phenomena to various immune stimulants were implicated [8, 9]. As more cases have been described, a correlation with various autoimmune diseases, viral hepatitis, and other concomitant cancers has become evident [8]. Autoimmune thyroiditis, primary biliary cirrhosis, Sjögren's syndrome, and other immunodeficiencies were found in patients with RLH, in addition to primary malignancies of the stomach, colon, kidneys, and ovaries [8, 10–12].

RLH is a histopathological diagnosis after surgical resection. The inaccuracy in preoperative diagnosis of RLH stems from the fact that these lesions are incidentally found on imaging studies done for other reasons, there is an absence of specific tumor markers, and last, a percutaneous biopsy of the inflammatory component would be nonspecific and the nature of the lesion is best determined through review of the overall morphology in the context of a resection specimen. Lesions are small and often misdiagnosed as malignant since they share features with hepatocellular carcinoma, specifically, being hypoechoic on ultrasound, hypodense on CT with variable enhancement characteristics, hypointense on T1-weighted and hyperintense on T2-weighted MRI [1, 13].

These similarities on imaging to HCC have sparked interest in further research into discovering any distinguishing features to help differentiate the two. There have been some reports of using “perinodular enhancement” as seen on RLH as a unique characteristic on CT, but this needs further investigation. In general clinical practice, a liver lesion workup is performed differently in different clinical backgrounds. Thus, in patients with no background liver dysfunction or cirrhosis or chronic viral hepatitis infection, RLH of the liver has to be indicated in the differential diagnosis, specifically when tumor markers like AFP are normal [1, 9].

These lesions can also be PET avid lesions raising the suspicion for metastatic disease to the liver; thus, appropriate metastatic workup has to be performed for tumors that most commonly spread to the liver [14].

**Figure 3:**
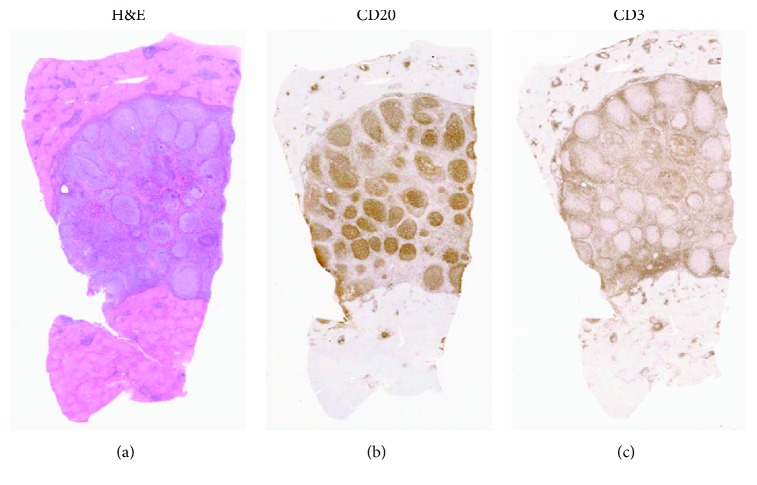
Hematoxylin-eosin stain of a section through the center of the nodule (a) shows numerous confluent lymphoid follicles composed of large, reactive lymphocytes with intervening smaller lymphoid cells. An immunostain for the B cell marker CD20 (b) shows that the follicles are composed of B cells, while the stain for the T cell marker CD3 (c) shows that the mantle zone and interfollicular areas have predominantly T cells, typical of benign lymphoid tissue.

**Figure 4:**
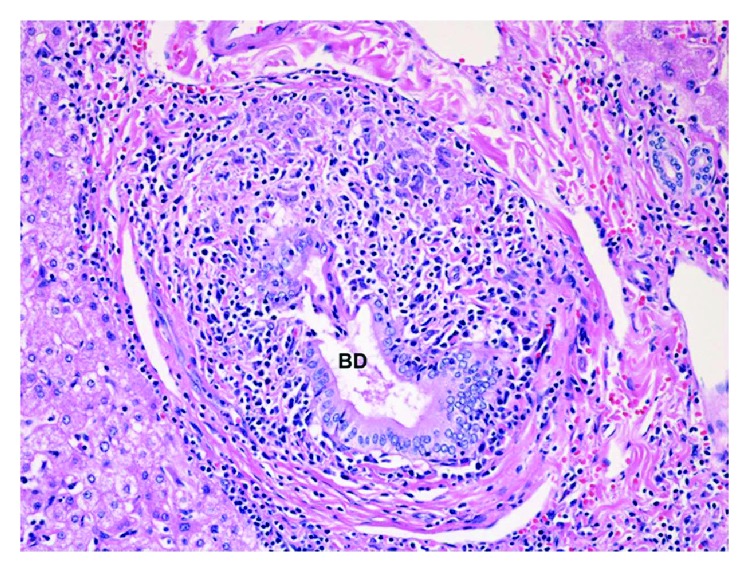
Portal area in the nonlesional liver tissue shows a severely injured bile duct (BD) infiltrated by chronic inflammatory cells and surrounded by ill-defined granulomatous inflammation, typical of the early stage of primary biliary cholangitis (formerly called primary biliary cirrhosis).

On pathology examination, Faris and Saltztein defined RLH as propagating lymphoid cell follicles without atypia, having reactive germinal centers [15]. It was also proposed that RLH is a localized, well-demarcated lesion among other tissues, with the presence of hyperplastic lymphoid follicles with polyclonal and polymorphic small mature lymphocytes, macrophages, and plasma cells in addition to surrounding stromal fibrosis [16]. Histologically, RLH of the liver has to be differentiated from inflammatory pseudotumors of the liver, low-grade lymphomas with nodular growth patterns such as follicular lymphoma and marginal zone lymphoma [1]. In our case, sections of the mass lesion (Figure 3) revealed a large nodule of hyperplastic benign lymphoid tissue composed of follicles of reactive B cells with normal mantle zones and interfollicular T cells. The liver tissue surrounding the lesion had considerable chronic lymphoplasmacytic portal inflammation. Many bile ducts in the inflamed portal tracts showed evidence of epithelial damage with infiltration by the inflammatory cells and surrounding granulomatous inflammation (Figure 4), typical of the destructive duct lesions of the autoimmune disease known as primary biliary cholangitis (formerly called primary biliary cirrhosis). This was decided to be Scheuer stage one, which is a known classification of PBC. As far as we can tell, only 30 cases of RLH of the liver associated with PBC have been reported in the English literature. Additionally, some of these cases may represent low-grade MALT lymphoma in the liver and may recur in other sites years after presentation; a diagnosis of MALT lymphoma needs to be excluded by ruling out clonality in the B cells for which immunohistochemical analysis or flow cytometric analysis is not sufficient, and a PCR analysis on the paraffin-embedded tissue block is warranted.

Finally, it is known from the literature that no cases of recurrence after resection were found on follow-up imaging, and no cases have been treated with anything additional to a surgical resection; thus, its natural history has yet to be defined [1, 17]. However, there is one case where a RLH of the liver degenerated into a low-grade lymphoma; the jury is still out on whether this was a lymphoma on the initial diagnosis [18]. RLH of other organs have been reported to have degenerated into lymphomas [9, 19].

## 4. Conclusion

Reactive lymphoid hyperplasia of the liver is a very rare disease of the liver, often found incidentally and often incorrectly diagnosed as hepatocellular carcinoma on preoperative workup. It has a predilection to middle-aged females, often associated with autoimmune diseases, specifically primary biliary cholangitis, or other malignancies. The definitive diagnosis, despite major advances in percutaneous biopsies and axial imaging techniques, remains a surgical resection diagnosis for appropriate morphological, immunohistochemical, and molecular studies. Patients with RLH of the liver have to be followed up closely after resection due to the rare incidence of malignant transformation.
